# Diversity of marine bacteria growing on leachates from virgin and weathered plastic: Insights into potential degraders

**DOI:** 10.1111/1758-2229.13305

**Published:** 2024-06-23

**Authors:** Cristina Romera‐Castillo, Stéphanie Birnstiel, Marta Sebastián

**Affiliations:** ^1^ Instituto de Ciencias del Mar‐CSIC Barcelona Spain

## Abstract

Plastic debris in the ocean releases chemical compounds that can be toxic to marine fauna. It was recently found that some marine bacteria can degrade such leachates, but information on the diversity of these bacteria is mostly lacking. In this study, we analysed the bacterial diversity growing on leachates from new low‐density polyethylene (LDPE) and a mix of naturally weathered plastic, collected from beach sand. We used a combination of Catalysed Reporter Deposition‐Fluorescence In Situ Hybridization (CARD‐FISH), BioOrthogonal Non‐Canonical Amino acid Tagging (BONCAT), and 16S rRNA gene amplicon sequencing to analyse bacterioplankton‐groups specific activity responses and the identity of the responsive taxa to plastic leachates produced under irradiated and non‐irradiated conditions. We found that some generalist taxa responded to all leachates, most of them belonging to the Alteromonadales, Oceanospirillales, Nitrosococcales, Rhodobacterales, and Sphingomonadales orders. However, there were also non‐generalist taxa responding to specific irradiated and non‐irradiated leachates. Our results provide information about bacterial taxa that could be potentially used to degrade the chemicals released during plastic degradation into seawater contributing to its bioremediation.

## Graphical abstract

Plastic degradation in the ocean leads to the release of chemical compounds that can be toxic to marine fauna. In this work, using a combination of BONCAT, CARD‐FISH, and 16S rRNA gene amplicon sequencing, we study the marine bacterial taxa that could potentially degrade such plastic leachates.

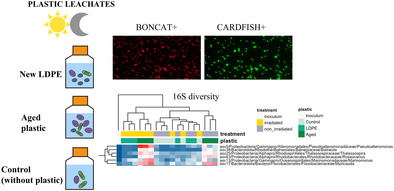

## INTRODUCTION

Plastic debris is giving rise to multiple problems for marine life and ecosystems. Many animals get entangled, while others ingest it, resulting in plastic entering the trophic web and potentially affecting humans. The research community is thus focusing on finding solutions to end plastic pollution through methods such as biodegradation. Identifying bacteria or enzymes able to degrade plastic has been the target of many studies in the last few years (Ru et al., 2020). However, plastic carries another problem that may be even worse than that caused by the polymer *per se*, as it is not composed of a single polymer, but incorporates a diverse plethora of chemicals that are added to provide the final material with the properties required for its intended use. Up to 60%–70% of plastic can be additives such as flame retardants, stabilizers, antioxidants, and others (Hahladakis et al., 2018; Hermabessiere et al., 2017). Plastic additives are bonded through weak forces such as Van der Waal so they can easily migrate to the aquatic media during plastic degradation (Zhang & Chen, 2014). These additives together with degradation products of the polymer are referred to as plastic leachates and can be toxic for many marine species. For example, leachates from high‐density polyethylene (HDPE) and polyinyl chloride (PVC) have been found to inhibit the growth and photosynthetic capacity of the cyanobacteria *Prochlorococcus* (Tetu et al., 2019). Likewise, leachates from PVC have been reported to increase antibiotic resistance and virulence in marine bacterial communities (Vlaanderen et al., 2023). The sea urchin *Paracentrotus lividus* exposed to leachates from PVC and beach‐collected pellets showed abnormalities including developmental delay, malformation of skeletal structures and nervous and immune systems, as well as abnormal axis formation (Rendell‐Bhatti et al., 2021). In addition, plastic leachates significantly inhibited barnacle *Amphibalanus amphitrite* cyprids settlement on glass (Li et al., 2016) and were toxic for *Daphnia magna* (Lithner et al., 2009) and the marine copepod *Nitocra spinipes* (Bejgarn et al., 2015). Plastic additives can also enter the trophic web, eventually reaching humans (Mamun et al., 2023). Therefore, besides preventing plastic from reaching the ocean, it is crucial to find microorganisms that can degrade both the polymers and their leachates.

When plastic leachates are released into seawater they become part of the dissolved organic carbon (DOC) pool, a process that is enhanced by sunlight radiation (Romera‐Castillo et al., 2018; Romera‐Castillo, Birnstiel, et al., 2022; Romera‐Castillo, Mallenco‐Fornies, et al., 2022). If the plastic is weathered and aged, the leaching is even higher (Romera‐Castillo, Birnstiel, et al., 2022). All that dissolved organic matter (DOM) released by plastic can alter the biogeochemistry of the water, such as decreasing its pH (Romera‐Castillo et al., 2023) or changing the composition of the DOM already present in the seawater.

Plastic leachates have been found to fuel marine bacterial metabolism, which means they can be degraded (Romera‐Castillo et al., 2018; Romera‐Castillo, Birnstiel, et al., 2022; Romera‐Castillo, Mallenco‐Fornies, et al., 2022; Zhu et al., 2020). Bacterial communities exposed to leachates from different plastic types, especially those exposed to sunlight radiation, presented higher single‐cell protein synthesizing activity than the control with no leachates (Birnstiel et al., 2022; Romera‐Castillo, Birnstiel, et al., 2022). The marine community growing on plastic leachates was found to be mainly composed of *Alteromonadales* (Gammaproteobacteria), followed by *Roseobacter* (Alphaproteobacteria) and unclassified Gammaproteobacteria (Birnstiel et al., 2022). However, that study was done using CARDFISH, with poor phylogenetic resolution, and there is little information about the identity of bacterial taxa degrading plastic leachates in seawater.

Recent work addressed the taxonomic composition of the bacterial community responding to low‐density polyethylene (LDPE) leachates in water from lakes (Sheridan et al., 2022). LDPE is the most commonly produced polymer worldwide and the most abundant in the ocean (Cózar et al., 2014; Suaria et al., 2016). However, until now, the only work analysing marine bacterial diversity growing on plastic leachates used PVC (Focardi et al., 2022). Here, we aimed to evaluate the taxonomic composition of marine bacteria thriving in leachates released from new LDPE, and from a mix of naturally weathered plastic collected from beach sand, which released large amounts of leachates (Romera‐Castillo, Birnstiel, et al., 2022). We analysed the level of activity of different bacterial groups that responded to the leachates using a combination of Catalysed Reporter Deposition‐Fluorescence In Situ Hybridization (CARDFISH) and BioOrthogonal Non‐Canonical Amino acid Tagging (BONCAT) and used 16S rRNA gene amplicon sequencing to address the identity of the responsive taxa. This knowledge will give us information about bacterial taxa that could be potentially used to remove the chemicals released into seawater during plastic degradation contributing to its bioremediation.

## EXPERIMENTAL PROCEDURES

### Plastic leachates preparation

Plastic leachate production is reported by Romera‐Castillo, Birnstiel, et al. (2022). In brief, low‐density polyethylene (LDPE, 0.5 mm thickness, Goodfellow) pieces and a mix of aged plastic collected in Famara Beach (Lanzarote, Spain) were used in this study. LDPE pieces were 5 × 5 mm in size, and the mix of aged plastic was composed of irregular flat pieces of around 0.7 cm average size. The aged mix was composed of 65% polyethylene, 30% polypropylene, and 5% polyvinyl chloride (Romera‐Castillo, Birnstiel, et al., 2022). A 2.48 g of new LDPE and ~7.5 g of a mix of aged plastic pieces were added to 500 mL quartz tubes filled with 400 mL of 0.2 μm filtered and sterilized aged seawater collected at 2000 m depth in the NW Mediterranean Sea and aged in the dark for 2 years. Non‐irradiated treatments were placed in 500 mL borosilicate bottles wrapped with aluminium foil. Controls with aged seawater without plastics were also incubated in irradiated and non‐irradiated conditions. The treatments were incubated for 6 days in a solar simulator placed in a flow‐through water bath at 28°C. The solar simulator provided artificial photosynthetic active radiation (PAR) by an HQI‐T Powerstar lamp (250 W, Osram), UV‐A radiation by 2 Philips TL100W/10 R fluorescent tubes, and UV‐B radiation by 2 UVA‐340 fluorescent lamps (Q‐Panel Company, UK). The radiation intensity for each wavelength or wavelength range was as follows: PAR (400–700 nm), 700 μmol m^−2^ s^−1^; 380 nm, 28.47 μW cm^−2^ nm^−1^; 340 nm, 16.31 μW cm^−2^ nm^−1^; 320 nm, 7.95 μW cm^−2^ nm^−1^; 305 nm, and 1.09 μW cm^−2^ nm^−1^. The radiation dose rate represents the solar radiation in the subtropical North Atlantic Gyre measured at noon at 15 m depth. Artificial solar radiation was measured at 305, 320, 340, 380 nm, and PAR with a Biospherical PUV‐510 radiometer. The irradiated treatments received continuous artificial solar radiation. At the end of the incubation, plastic pieces were removed, and the treatments with aged plastic were filtered through a pre‐combusted (4 h, 450°C) Whatman GF/F filter since some plastic particles were observed in the water.

### Microbial community degrading plastic leachates

All the treatments were inoculated with surface seawater collected from the Blanes Bay Microbial Observatory, in the coastal NW Mediterranean Sea (41°40′N, 2°48′E). This surface seawater was filtered by 0.8 μm to remove grazers and other eukaryotic cells, since they could mask the prokaryotic response to leachates, and was inoculated into the leachates/control samples at a ratio of 9:1 (leachate: inoculum) on the same day the water was collected (January 30th, 2020). The treatments were amended with NH_4_Cl and NaH_2_PO_4_ to a final concentration of 10 and 2 μmol mL^−1^, respectively, to avoid growth limitation by either nitrogen or phosphorus availability. The treatments were incubated in the dark at 23°C until the prokaryotic community reached the stationary phase on day 4 of the experiment (see Figure S1). Samples for prokaryotic abundance were collected daily and analysed through flow cytometry to follow the growth curve as detailed in Figure S1. The final cell abundance values reached in each treatment are shown in Table 1. Samples for CARD‐FISH analyses and BONCAT were collected at 24 h (t1) and 96 h (t4) after the inoculation.

**TABLE 1:** Total DOC and integrated FDOM in the protein‐ and humic‐like region of the plastic leachates and controls (i.e., the different treatments at the beginning of the degradation experiment, T0) and bacterial abundance at the end of the experiment (TF).

Plastic type	Treatment	Total DOC T0 (umol L^−1^)	FDOM protein‐like T0 (R.U. nm^2^)	FDOM humic‐like T0 (R.U. nm^2^)	Bacterial abundance TF (10^6^ cell mL^−1^)
LDPE film	Irrad	94.50 ± 0.33	51.67 ± 2.65	64.57 ± 2.80	3.15 ± 0.31
	Non‐irrad	79.56 ± 0.2	50.54 ± 3.87	117.71 ± 3.26	2.78 ± 0.19
Aged mix	Irrad	665.87 ± 1.70	972.84 ± 54.76	309.66 ± 22.99	13.4 ± 2.75
	Non‐irrad	492.53 ± 1.66	1695.00 ± 54.36	573.92 ± 21.46	9.79 ± 0.88
Control	Irrad	81.36 ± 0.12	41.75 ± 3.57	71.98 ± 1.81	3.06 ± 0.34
	Non‐irrad	73.62 ± 0.35	45.21 ± 3.02	110.56 ± 2.73	2.71 ± 0.23

*Note*: The treatments include leachates produced by virgin LDPE and the aged plastic mix and controls with no plastic that were maintained under the same conditions during the leaching process (see Romera‐Castillo, Birnstiel, et al., 2022 for more details).

### Initial DOC concentration and leachates characterization

The amount of organic carbon present in the different treatments after the inoculation of surface water was measured at the beginning of the experiment using a Shimatzu TOC‐V organic carbon analyser as detailed in (Romera‐Castillo, Birnstiel, et al., 2022). Leachates were further characterized based on the optical properties of their fluorescent fraction using an LS 55 PerkinElmer Luminescence spectrometer (see Romera‐Castillo, Birnstiel, et al. (2022) for more details).

### DNA extraction and 16S rRNA gene amplicon sequencing

Samples (265–285 mL) for DNA extraction were collected from the inoculum at the beginning and the end of the plastic leachate degradation experiment (on day 4). DNA extractions were performed using the standard phenol‐chloroform protocol with slight modifications (Logares et al., 2014). Samples were sent for sequencing at the Research and Testing Laboratory (Lubbock, TX, USA). We used the primers 515F and 926‐R (Parada et al., 2016) to amplify the V4‐V5 region of the 16S rRNA gene. Illumina MiSeq 2 × 300 flow cells were used for sequencing following Illumina protocols described elsewhere (Cúcio et al., 2016).

Exact amplicon sequence variants were obtained with DADA2 v1.8 (Callahan et al., 2016). Primers and spurious sequences were trimmed using cutadapt (Martin, 2011). We run it twice using the following parameters: –pair‐filter = any –interleaved –minimum‐length = 32. DADA2 v1.8 was used to differentiate exact sequence variants (Callahan et al., 2016) with the following parameters: *trunclen* = (250, 180), *maxEE* = (2, 4), *minOverlap* = 15, pool = TRUE. After filtering through DADA2 and the chimera check, 50% of the rDNA reads were retained for further analyses. Taxonomic assignment was performed using the DADA2 function ‘assignTaxonomy’ against SILVA v.138 (Quast et al., 2013).

Different bacteria contain different 16S gene copy numbers, and this may affect the relative abundance of the groups observed in 16S sequencing data. To avoid this bias, the relative abundance of each ASV was corrected by their average 16S rRNA gene copy number using the tool ‘estimate’ of the ribosomal RNA database (Stoddard et al., 2015). For this calculation, we took into account the taxonomic resolution available for each ASV and used the estimated value for the lowest available taxonomic rank (order, family, or genus, Table S1), as the average gene copy number is quite conserved among closely related taxa. When the lowest available taxonomy rank for the ASVs was Class, we did not correct for 16S rRNA copy number as its inaccurate prediction may introduce a larger bias in community composition (Louca et al., 2018).

### Data treatment and statistical analyses

All data treatment and statistical analyses were conducted with the R Statistical Software using version 4.0.0.

Prokaryotic richness (the number of ASVs per sample) and sample evenness (using the Pielou index (*J* = *H*/ln(nASV), where *H* is the Shannon index and nASV is the richness in every sample)), were calculated using the ‘vegan’ 2.5–7 package after rarefaction to the minimum number of reads (4436 reads) using the *rrarefy* function in the ‘vegan’ package (process was repeated 100 times and the mean number of reads (rounded to integers) from the 100 rarefactions was used). No samples were removed in this process.

Permutational multivariate analysis of variance (PERMANOVA) was conducted with 1000 permutations to test for significant differences in the taxonomic composition between groups of samples using the interactions of ‘plastic’ (i.e. aged, LDPE, or control) and ‘light treatment’ (irradiated or non‐irradiated) as the grouping variables.

The heatmap to visualize the response of the most abundant ASVs (those with relative abundances >1% in at least one sample) in the different treatments was done using the ‘pheatmap’ package version 1.0.12 (Kolde, 2019). A hierarchical clustering was carried out based on Euclidean distances of centred‐log‐ratio transformed ASV abundance tables (Jones & Aitchison, 1987) to group ASVs with similar behaviours. The minimum number of clusters showing contrasting behaviours in the different treatments and types of leachates was chosen by visual inspection, resulting in seven clusters. The relevance of each of these clusters in the different leachates was further explored by looking at their contribution to the total community in each of the treatments.

Statistical differences between treatments were tested using Kruskal−Wallis and Dunn post hoc tests (performed using the ‘fsa’ package version 0.9.5 (Ogle et al., 2023)). A *p*‐value <0.05 was considered significant.

### Single‐cell protein synthesizing activity of different taxonomic groups

The response to plastic leachates of different taxonomic groups was analysed through a combination of CARD‐FISH and BONCAT (Dieterich et al., 2006). Samples were collected on day 1 and day 4 of incubation. Nine‐mL samples were incubated with the amino‐acid surrogate L‐Homopropargylglycine (HPG) (final concentration 1 μM) for 2.5 h. Incubations were performed in triplicate. Then, samples were fixed with 0.2 μm‐filtered paraformaldehyde (PFA; final concentration 4% [v/v]) overnight at 4°C. Samples were then filtered through a 0.2 μm pore size polycarbonate filter on top of a 0.8 μm cellulose acetate membrane filter, and filters were rinsed 3 times with sterile MiliQ water and stored at – 80°C until further processing. After thawing, the filters were dipped in a previously boiled 0.1% (w/w) low‐gelling‐point agarose solution to attach the cells to the filter and prevent cell loss during permeabilization and downstream procedures, then dried at 37°C, and dehydrated with 95% ethanol. Bacterial cells were permeabilized with lysozyme (10 mg ml^−1^; 0.05 M EDTA, 0.1 M Tris–HCL, 1 h) and achromopeptidase (60 U ml^−1^; 0.01 M Na Cl, 0.01 M Tris–HCl, pH 7.6, 30 min) at 37°C, following standard protocols (Sekar et al., 2003). The filters were then cut into different sections and CARD‐FISH was performed following the protocol detailed in (Pernthaler et al., 2002). The filter sections were hybridized with the following horseradish peroxidase (HRP)‐labelled probes: GAM42a together with its unlabelled competitor probe, which targets most Gammaproteobacteria (Manz et al., 1992), Alf968, which targets most Alphaproteobacteria (Neef, 1997), CF319a, which targets Flavobacteriales, Cytophaga and other members of the Bacteroidetes phylum (Manz et al., 1996), EUB338 I‐II and –III, which targets most Bacteria (Daims et al., 1999), ROS537, which targets *Roseobacter* (Eilers et al., 2001), and ALT1413, which targets Alteromonadales (Eilers, Pernthaler, Glöckner, & Amann, 2000). Specific hybridization conditions were established by the addition of formamide to the hybridization buffers (60% formamide for the ALT1413 probe, 45% for the Alf968, and 55% for the other probes). Hybridization was performed overnight at 35°C. For amplification, a tyramide labelled with Alexa488 was used.

BONCAT click chemistry reaction was performed after the CARDFISH hybridization, to assess the contribution of each CARDFISH targeted bacterial group to the community activity. A detailed description of the protocol can be found in (Birnstiel et al., 2022).

Filters were counterstained with 4′,6‐diamidino‐2‐phenylindole (DAPI; final concentration 1 μg mL^−1^) and analysed by epifluorescence microscopy to quantify the single‐cell protein synthesizing activity of each targeted phylogenetic group. Details on image acquisition and analyses can be also found in (Birnstiel et al., 2022). To calculate the BONCAT+ cells pictures were taken in black and white, three micrographs from the same field of view, one for DAPI, one for BONCAT and one for CARDFISH. To consider a cell BONCAT+ it needed to be DAPI+. The fluorescence intensity of the BONCAT+ cells was assessed using the mean grey value (MGV), which is the sum of the grey values of all the pixels in the cell divided by the number of pixels. This fluorescence intensity is proportional to the activity of the cells (Leizeaga et al., 2017). Thus, the summed intensity of the BONCAT signal in each sample divided by the number of images (fields of view; FOV) taken for that sample, was used as an estimation of the total activity of the cells in the sample. The contribution of each CARDFISH targeted group to activity (i.e. BONCAT signal intensity) was calculated for each sample by dividing the sum of the MGV of the BONCAT signal of each CARDFISH+ probe by the sum of the MGV of all BONCAT+ cells in the sample.

## RESULTS

### Contribution of different bacterioplankton groups to the bulk community response to plastic leachates

**FIGURE 1:**
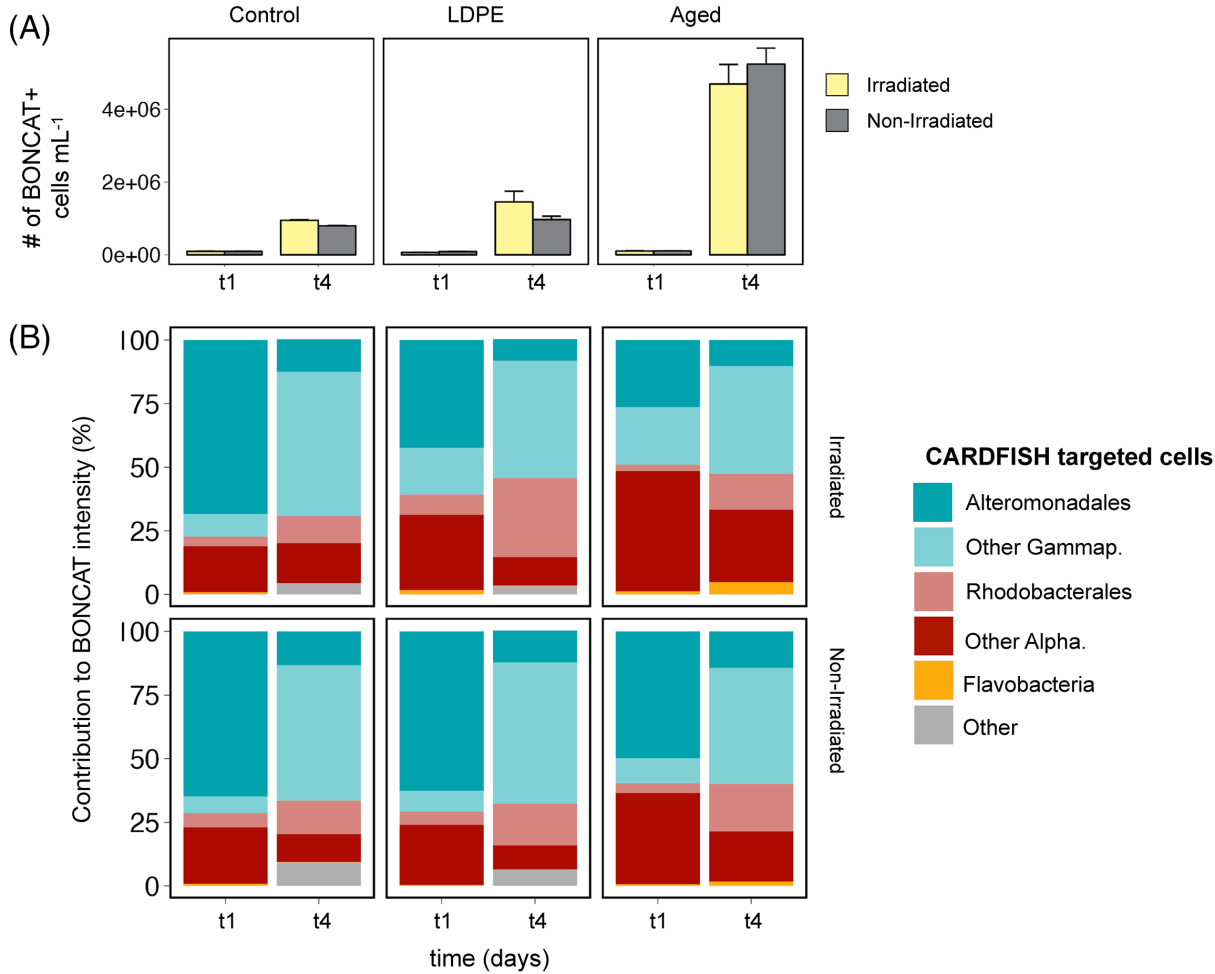
Single‐cell activity response to plastic leachates estimated using a combination of BONCAT‐CARDFISH. (A) Number of BONCAT‐positive cells in each of the treatments at day 1 (t1) and day 4 (t4). (B) Contribution of the different targeted groups to the total BONCAT intensity (as a proxy for contribution to activity). Other Gammaproteobacteria represent Gammaproteobacteria that are not labelled by the Alteromonadales probe (i.e. total Gammaproteobacterial cells minus Alteromonadales cells). Other Alphaproteobacteria represent Alphaproteobacteria excluding Rhodobacterales, and the Other category represents prokaryotes that are BONCAT+ and DAPI+ but not labelled by any of the CARDFISH probes used. Control (with no plastic leachates), LDPE: low‐density polyethylene leachates, Aged: aged plastic collected from beach sand. Irradiated denotes those leachates produced under simulated solar light, whereas non‐irradiated refers to those leachates produced under dark conditions (see material and methods and Romera‐Castillo, Birnstiel, et al., 2022 for details).

Compared to commercial LDPE, aged plastic leached substantially higher concentrations of DOC, characterized by a marked signature of protein‐like FDOM (Table 1, Romera‐Castillo, Birnstiel, et al., 2022). This led to a significant stimulation of heterotrophic bacteria in the aged plastic leachates, which reached cell abundance values of 1.34 × 10^7^ and 9.79 × 10^6^ cell mL^−1^ in the irradiated and non‐irradiated treatments, respectively (Table 1). Likewise, the number of active (BONCAT+) bacteria was also markedly higher in the aged plastic leachates than in the rest of the treatments (Figure 1A). Using BONCAT in combination with CARD‐FISH, we investigated the bacterioplankton groups that were responsible for the changes in the abundance and activity of bacteria in the different treatments (Figure 1A). We targeted the groups Alphaproteobacteria, Gammaproteobacteria, and Flavobacteria, as they typically dominate heterotrophic bacterial communities of the surface ocean (Barberan & Casamayor, 2010). Additionally, we focused on Alteromonadales and Roseobacter, within the Gammaproteobacteria and Alphaproteobacteria classes, respectively, as they are known to be among the first responders to organic carbon inputs (Allers et al., 2007). Despite the difference in the magnitude of the response, most of the activity in all treatments was linked to Gammaproteobacteria (Alteromonadales and other unidentified Gammaproteobacteria) and Alphaproteobacteria (Rhodobacterales and unidentified Alphaproteobacteria) (Figure 1B). Alteromonadales dominated the activity on day 1 in non‐irradiated LDPE and aged plastic leachates, as well as in both the irradiated and non‐irradiated control samples. In contrast, in irradiated aged plastic leachates, the contribution of Alteromonadales decreased and, instead, unidentified Gammaproteobacteria and Alphaproteobacteria were responsible for the activity (Figure 1B). On day 4, at the stationary growth phase (Figure S1 and Romera‐Castillo, Birnstiel, et al., 2022 for details), the contribution of Alteromonadales decreased in all treatments, and unidentified Gammaproteobacteria and Rhodobacterales became more active (Figure 1B). The contribution of Rhodobacterales to activity was particularly high in the irradiated LDPE plastic leachates. In the irradiated aged plastic leachates, there was some contribution to the activity of Flavobacteria, which were practically inactive in the other treatments (Figure 1B).

### Shifts in the diversity of prokaryotic communities growing on plastic leachates

**FIGURE 2:**
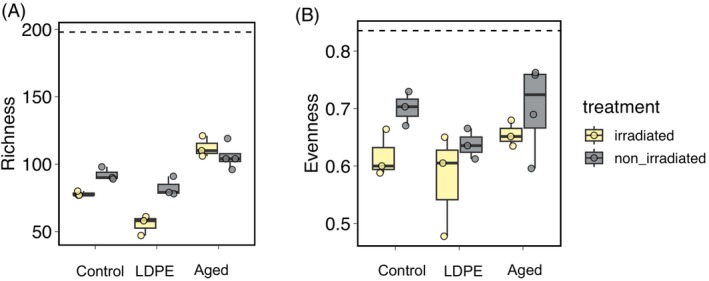
Shifts in taxonomic richness and evenness in prokaryotic communities growing on the plastic leachates on day 4, compared to the initial inoculum (dashed line). Control (no plastic leachates), LDPE: low‐density polyethylene leachates, plastic collected from beach sand. Irradiated denotes those leachates produced under simulated solar light, whereas non‐irradiated refers to those leachates produced under dark conditions (see material and methods and Romera‐Castillo, Birnstiel, et al., 2022a for details).

In terms of diversity indexes, there was a notable decrease in both the richness and evenness of prokaryotic communities compared to the original communities (i.e. inoculum, Figure 2) in all treatments (control, LDPE, and aged plastic leachates). This decrease was usually higher (although not statistically significant) in the irradiated treatments, indicating a trend in which non‐irradiated treatments sustained communities that were more diverse and less dominated by particular taxa than the irradiated ones. This effect was more pronounced in the LDPE leachates (Figure 2A,B). Aged plastic leachates showed slightly higher species richness than the LDPE treatments, although differences with the control were not significant (Kruskal–Wallis rank sum test and Dunn post hoc, *p* > 0.05). Species richness was correlated with the proportion of protein‐like DOM (in relation to total DOC, see Table 1) at the beginning of the biodegradation experiment after the leaching process (Spearman R = 0.74, *p* < 0.0001, see Table 1 for initial FDOM and DOC characteristics).

### Bacterial communities growing on plastic leachates

**FIGURE 3:**
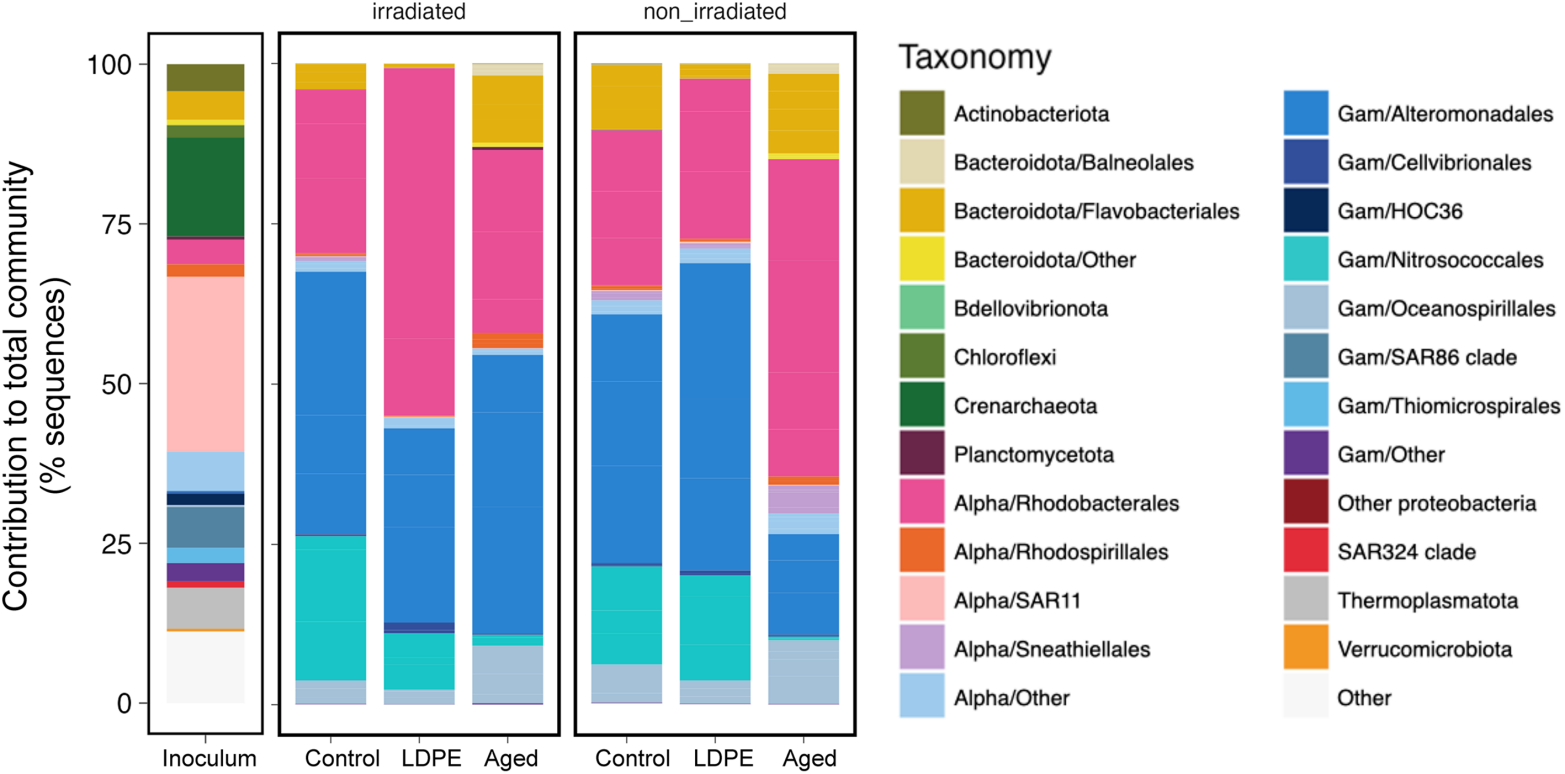
Taxonomic composition of the prokaryotic communities in the inoculum, in the different plastic leachates and the control for the irradiated and the non‐irradiated treatments at the end of the experiment (day 4). Taxonomic affiliation is shown at the phylum level, except for Bacteroidota and the Proteobacteria classes Alphaproteobacteria and Gammaproteobacteria, which are shown at the order level. Inoculum: taxonomic composition of the surface seawater sample used as inoculum for the different treatments. Control (with no plastic leachates), LDPE: low‐density polyethylene leachates, Aged: aged plastic collected from beach sand. Irradiated denotes those leachates produced under simulated solar light, whereas non‐irradiated refers to those leachates produced under dark conditions (see material and methods and Romera‐Castillo, Birnstiel, et al., 2022 for details).

The observed changes in alpha diversity were accompanied by changes in community composition from the inoculum to the experimental treatments (Figure 3). Taxonomic groups that dominated the inoculum, such as SAR11 and Crenarchaeota, disappeared in the experimental treatments. The type of plastic (i.e. either aged or LDPE plastic leachates, or control samples without plastic) explained 36% of the total variance in the community composition on day 4 (PERMANOVA, *p* = 0.0019), whereas the light treatment (i.e. irradiated vs non‐irradiated) explained only 9% (*p* = 0.025), and their interaction 16% of the variance (*p* = 0.026). These findings indicate that communities were significantly different in the different leachates and the control. At coarse taxonomic resolution, the composition in the control, LDPE, and aged plastic leachates was similar in both the irradiated and non‐irradiated treatments, with a dominance of Rhodobacterales and Alteromonadales (Figure 3). Rhodobacterales represented >50% of the sequences in both the irradiated LDPE treatment and the non‐irradiated aged plastic leachates, whereas their contribution in the rest of the treatments was remarkably lower (~25% of the sequences, Figure 3). These treatments were instead dominated by Alteromonadales. Nitrosococcales (Gammaproteobacteria) were overrepresented in the control and the LDPE leachates (particularly in the non‐irradiated ones), but their contribution to the communities growing on aged plastic leachates was negligible. Oceanospirillales (Gammaproteobacteria) increased notably in relative abundance (~10% of the reads) in both the irradiated and non‐irradiated aged plastic leachates compared to the Control and the LDPE leachates (Figure 3). Aged plastic leachates contained a notable fraction of Flavobacteriales when compared with LDPE and control, particularly in the irradiated treatments.

**FIGURE 4:**
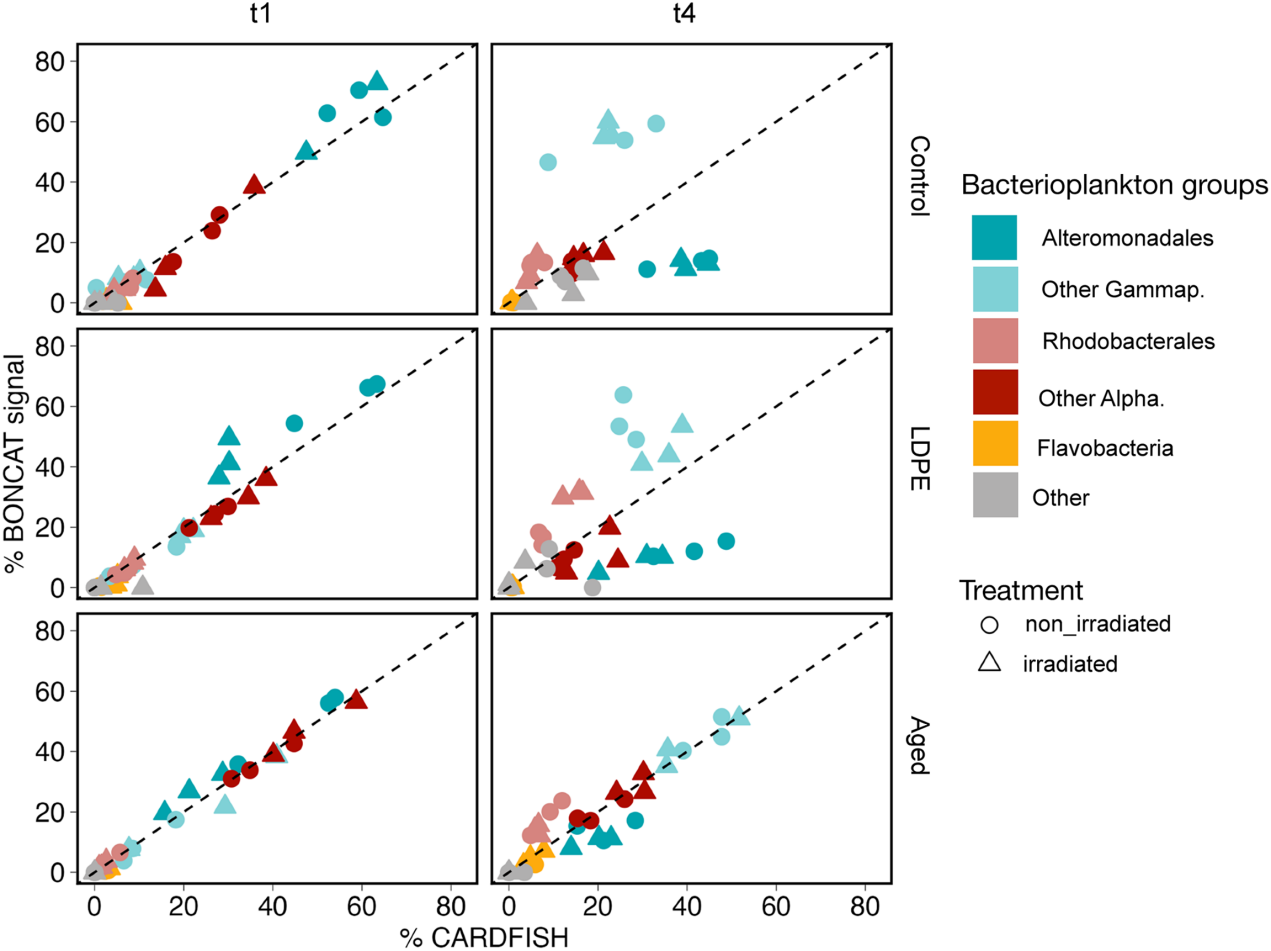
The relative contribution of specific bacterioplankton groups to the bulk bacterial abundance determined by CARD‐FISH versus their contribution to activity assessed through BONCAT signal intensity on day 1 (left panel) and day 4 (right panel) of the experiment. Circles denote the non‐irradiated treatments and triangles the irradiated treatments. The colour indicates the different bacterioplankton groups considered. The dashed lines indicate a 1:1 relationship.

The dominance of Alteromonadales and Rhodobacterales in the 16S RNA gene‐based communities across all treatments contrast with the BONCAT‐CARDFISH results (Figure 1), which showed that on day 4 Alteromonadales represented a small fraction of the active Gammaproteobacteria. Likewise, BONCAT‐CARDFISH analyses revealed that Alphaproteobacteria, other than Rhodobacterales, played an important role in the activity. This lack of agreement may be partially related to the fact that 16S rRNA gene sequences do not differentiate active from inactive cells, so the high abundance of Alteromonadales may reflect past activities, that is the stimulation of this group in the early days of the experiment and their subsequent inactivation following the exhaustion of their preferred compounds. To explore this, we compared the relative contribution of the different bacterioplankton groups to bulk bacterial abundance (estimated through CARDFISH) and their contribution to activity assessed through the BONCAT signal intensity on both day 1 and day 4 of the experiment (Figure 4). On day 1 of the experiment, the contribution of the different groups to total bacterial abundance was largely proportional to their contribution to community activity (Figure 4), which is typical for a community that is actively growing. Conversely, on day 4 of the experiment, some groups like Alteromonadales contributed less to the activity than to the cell abundance, particularly in the control and LDPE leachates (Figure 4), suggesting this group responded very quickly at the beginning but afterwards got inactivated throughout the experiment. The overrepresentation of gammaproteobacteria other than Alteromonadales in the active pool of cells on day 4 indicates that these bacteria got stimulated at the end of the experiment. Nonetheless, other factors such as varying hybridization efficiency of the CARD‐FISH probes may also play a role in the discrepancy observed between sequencing data and the BONCAT‐CARDFISH data, as discussed later.

### Identity of the most responsive taxa

**FIGURE 5:**
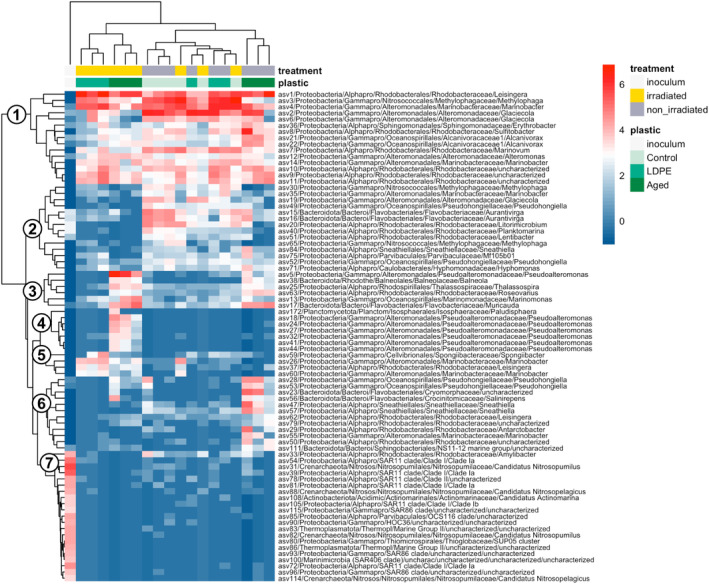
Heatmap showing the most abundant taxa (ASVs representing >1% in relative abundance) in the leachates degradation experiment. Upper color bars represent the inoculum, and the irradiated and non‐irradiated treatments for the control, the LDPE, and aged plastic leachates. Rows represent the different ASVs with their corresponding taxonomic affiliation down to the genus level. The colour key represents the abundance (centred‐log‐ratio normalized DNA reads) of each of the ASVs, with the red colour representing higher abundances. Numbers on the left indicate the different clusters of responsive taxa identified upon a hierarchical clustering of ASVs with similar trends based on Euclidean distances. Control (no plastic leachates), LDPE: low‐density polyethylene leachates, Aged: aged plastic collected from beach sand. Irradiated denotes those leachates produced under simulated solar light, whereas non‐irradiated refers to those leachates produced under dark conditions (see material and methods and Romera‐Castillo, Birnstiel, et al., 2022 for details).

**FIGURE 6:**
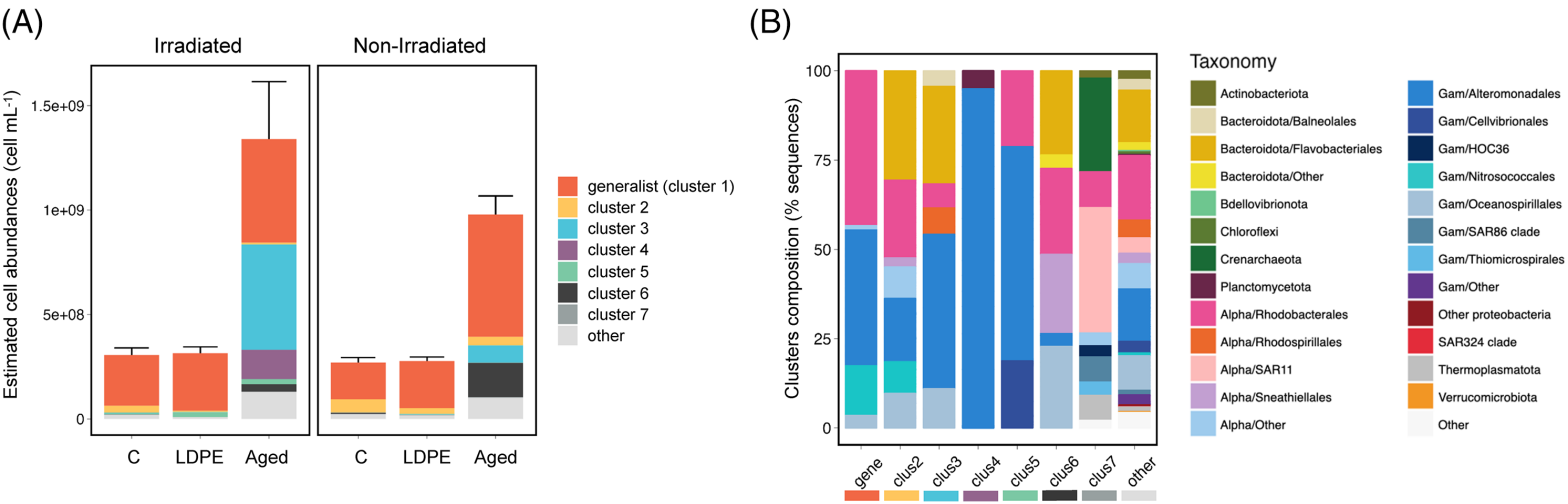
(A) Contribution of the different clusters delineated in Figure 5 to cell abundances in each of the treatments on day 4 of the experiment (estimated by multiplying the proportion obtained by sequencing by total cell counts estimated by flow cytometry). The error bar represents the standard deviation of total cell abundance for the three replicates. Colors represent the different clusters. The ‘other’ category indicates those ASVs that were always rare (below 1% relative abundance in all samples). (B) Taxonomic composition (in terms of sequences) of each cluster (abbreviated as ‘clus’, except for the generalist cluster that is named ‘gene’. The colour bar at the bottom indicates the different clusters. Control (no plastic leachates), LDPE: low‐density polyethylene leachates, Aged: aged plastic collected from beach sand. Irradiated denotes those leachates produced under simulated solar light, whereas non‐irradiated refers to those leachates produced under dark conditions (see material and methods and Romera‐Castillo, Birnstiel, et al., 2022 for details).

Despite the taxonomic composition among the different treatments was rather homogenous at coarse phylogenetic resolution, differences arose when explored at the taxon‐specific level (Figure 5). Hierarchical clustering based on Euclidean distances allowed the grouping of ASVs in different clusters based on similar responses to the plastic leachates (Figure 5). There were some generalist taxa (cluster 1, Figure 5) that belonged to the Alphaproteobacteria Rhodobacterales and Sphingomonadales, and the Gammaproteobacteria Alteromonadales, Oceanospirillales and Nitrosococcales. These generalist taxa grew in most of the treatments, and together represented between ~40% in the irradiated aged plastic leachates to 80%–90% in the irradiated control and LDPE leachates (Figure S2), although their estimated cell abundance was substantially higher in the aged plastic leachates (Figure 6). However, even though they accounted for a notable fraction of the community in most cases, some ASVs displayed certain preferences for the different leachates. For example, Rhodobacterales asv1 (assigned as *Leisingera methylohalidivorans*, Table S1) represented on average 40% of the community in the irradiated LDPE leachates, but ~20% in both irradiated and non‐irradiated aged plastic leachates and between 5% and 10% of the community in the irradiated and non‐irradiated control (Figure S3). In contrast, the Nitrosococcales asv3 (*Methylophaga* sp., 99.73% sequence identity to *Methylophaga alcalica*, Table S1), which was also categorized as generalist, accounted on average for 25% of the reads in the irradiated control, and around 15% in the non‐irradiated LDPE leachates, but its contribution to the aged plastic leachates was notable lower (Figure S3).

Cluster 2 represented taxa that responded more to the non‐irradiated treatments than to the irradiated ones, particularly in the control, where they represented up to 25% of the community (Figure S2), and LDPE leachates, where they accounted for 10% of community sequences (Figures S2 and 6). These taxa had a diverse phylogenetic assignment, including Flavobacteriales (Bacteroidota), the Gammaproteobacteria Nitrosococcales, Alteromonadales, and Oceanospirillales, and the Alphaproteobacteria Rhodobacterales, Caulobacterales, Parvibaculales and Sneathiellales (Figures 5 and 6, Table S1). Cluster 3 comprised taxa that were largely stimulated in the irradiated aged plastic leachates, where they represented ~40% of the community (Figures S2 and 6). Their representation in the non‐irradiated aged plastic leachates was substantially lower (Figure 6), accounting for 10% of the community (Figure S2). Nonetheless, these taxa were negligible in the control and LDPE leachates. Cluster 3 taxa belonged to Alteromonadales (*Pseudoalteromonas shioyasakiensis*), Oceanospirillales (*Marinomonas posidonica*), Flavobacteriales, Balneolales, Rhodobacterales, and Rhodospirillales (see detailed taxonomy in Table S1). Cluster 4 contained taxa that exhibited a response specifically in the irradiated aged plastic leachates (where they represented ~12% of the community, Figure S2). These taxa did not show a similar response in the non‐irradiated aged plastic leachates, LDPE leachates, or control samples (Figure 6). Within this cluster there were 6 closely related ASVs associated with Pseudoalteromonas (Figure S4) and one Planctomycetes ASV (*Paludisphaera*) (Table S1). Cluster 5 contained 4 taxa that were responsive to the irradiated LDPE leachates (representing around 8% of the community, Figure S2) and belonged to Alteromonadales (*Marinobacter*), Cellvibrionales (*Spongiibacter*), and Rhodobacterales (*Leisingera methylohalidivorans*). Finally, cluster 6 comprised taxa that had a preference for the non‐irradiated aged plastic leachates (Figure 6), and accounted on average for 15% of the community (Figure S2). These taxa belonged to diverse phylogenetic groups, such as Alteromonadales (*Marinobacter*), Oceanospirillales (*Pseudohongiella nitratireducens*), Flavobacteriales (*Crocinitomicaceae* and *Cryomorphaceae*), Sphingobacteriales, Rhodobacterales (Leisingera, Antarctobacter and other unknown Rhodobacterales), and Sneathiellales (Table S1). Cluster 7 included taxa that were present in the inoculum but did not develop in any treatment (Figure 5, and Figure 6).

## DISCUSSION

### Response of bacterial communities to plastic leachates and methodological considerations

Our study shows that exposure of natural communities to plastic leachates led to a drastic change in activity and community structure, although we found also a notable change in community structure in the control treatment. This can be explained by the fact that the bacterial inoculum was prefiltered through 1 μm to remove predators, and then diluted 1:9 into either the control (seawater with no plastic leachates) or LDPE and aged plastic leachates (see methods). Removal of predators and the increase in per‐cell resources upon dilution usually lead to the development of fast growers (e.g. Fecskeová et al., 2021; Ferrera et al., 2011; Teira et al., 2019), resulting in remarkable changes in community composition (Fecskeová et al., 2021; Sánchez et al., 2020; Sebastián et al., 2021). Despite the changes observed across all treatments, bacterial communities exhibited a substantially higher response to the aged plastic leachates than to the control and LDPE leachates (Figures 1 and 6A). The blooming of fast growers was likely the cause of the decrease in community richness across all treatments (Figure 2). However, this decrease was comparatively lower in the aged plastic leachates, suggesting that these leachates favour the growth of a more diverse assemblage of taxa.

The combination of sequencing data and BONCAT‐CARDFISH provided insights into the dynamics of the response to the plastic leachates. While Alteromonadales and Rhodobacterales dominated the sequenced‐based communities across all treatments on day 4, the BONCAT‐CARDFISH analyses revealed that Alteromonadales were to a great extent inactive on day 4 (Figure 4), particularly in the control and LDPE leachates. This finding aligns with the observed decrease in the proportion of active cells on day 4 of the experiment, dropping from over 70% on day 1 to 30%–50% on day 4 (see Romera‐Castillo, Birnstiel, et al., 2022 for further details). Other factors such as low ribosomal content (Bouvier & Del Giorgio, 2003) or varying hybridization efficiency of the probes to the target cells could lead to a discrepancy between the CARDFISH labelling of the groups and the sequencing data. For example, the probe used here for Alteromonadales, ALT1413, targets mostly the genera *Alteromonas*, *Colwellia*, and *Glaciecola* (Eilers, Pernthaler, & Amann, 2000), and may not label other Alteromonadales genera like *Marinobacter* or *Pseudoalteromonas*, that responded to the plastic leachates (Figure 5). Despite the potential unspecificity of the probes, we generally found a good agreement between the cell abundance estimation of the different bacterioplankton groups by CARDFISH and 16S rRNA gene sequencing (Figure S5), except for the Rhodobacterales, which showed higher values in the 16S‐based communities than in the CARDFISH based, suggesting the inability of the probe to target all Rhodobacterales cells. Yet, considering the overall consistency observed with the rest of the probes, the difference noted between the 16S‐based community composition and the active communities likely reflects the inactivation of distinct members of the community during the length of the experiment.

Alteromonadales and Rhodobacterales often dominate prokaryotic communities upon experimental manipulation (e.g. Allers et al., 2007; Birnstiel et al., 2022), as they are groups that display very fast growth rates (Ferrera et al., 2011; Teira et al., 2009) and have been described as metabolically diverse opportunitrophs capable of taking advantage of a broad variety of organic compounds (Mena et al., 2022). This is probably the reason they dominated communities in both the control and plastic leachates (Figure 3). Nonetheless, although in terms of broad taxonomic composition the response seemed similar between the plastic leachates and the control, bacterial abundance reached one order of magnitude higher values in the aged plastic leachates in relation to the LDPE leachates and control treatment (Table 1, Figure 6 and Romera‐Castillo, Birnstiel, et al., 2022). This is likely a consequence of the large amount of DOM released by aged plastic in comparison to virgin LDPE (Romera‐Castillo, Birnstiel, et al., 2022), and the higher amount of protein‐like fluorescent DOM. Even if the plastic leachates do not contain amino acids or proteins, they present FDOM fluorescing in the protein‐like region which was observed to be labile to microbial uptake (Romera‐Castillo, Birnstiel, et al., 2022). Those protein‐like fluorescent compounds could be, for instance, polycyclic aromatic hydrocarbons which are known to fluoresce in that region and may be released by plastic (Barrero‐Moreno et al., 2018). Rhodobacterales and Alteromonadales are frequently found within plastic‐associated communities (Bhagwat et al., 2021; Debroas et al., 2017; Zettler et al., 2013), and are known for the ability to degrade complex compounds hydrocarbons and plasticizers (Buchan et al., 2019; Chronopoulou et al., 2015; Focardi et al., 2022; Vejarano et al., 2019). These plasticizers are plastic additives that are usually released to the environment during the degradation process (Paluselli et al., 2019) and were likely very abundant in the aged plastic leachates. Although some studies have shown that the highest amount of material is leached during the first hours in contact with water (Romera‐Castillo et al., 2018), long‐term studies observed that the leaching of aged plastic follows a linear relationship with time (Zhu et al., 2020). Also, we cannot exclude the release of persistent organic pollutants by aged plastic since in seawater, plastic can adsorb them (Bakir et al., 2014; Mato et al., 2001) and they may be subsequently released when the local environmental conditions change.

The change in the contribution of the different groups to the activity from day 1 to day 4 is consistent with a succession in the utilization of resources, as different taxa specialize in the utilization of specific compounds and some bacteria may rely on the metabolic byproducts of others (Pontiller et al., 2022; Sharma et al., 2014; Teeling et al., 2012). Alteromonadales usually are responsible for a large fraction of labile organic compounds consumption in the ocean (Pedler et al., 2014), and despite their described ability to use a broad diversity of compounds, their abundance usually decreases once these labile compounds are exhausted (Pedler et al., 2014; Sebastián et al., 2021).

### Identity of the bacterial taxa growing on plastic leachates

When looking at finer taxonomic resolution, we found that the response to leachates was largely driven by generalist taxa belonging to the Alteromonadales (*Alteromonadaceae* and *Marinobacteraceae*), Oceanospirillales (*Alcanivoracaceae*), Nitrosococcales (*Methylophagaceae*), *Rhodobacteraceae*, and *Sphingomonadaceae*. All these groups contain taxa that specialize in hydrocarbon degradation in the marine environment (Head et al., 2006).

Moreover, Alteromonadales has been reported to grow in the presence of PVC leachates in a recent study where 60 μm filtered seawater containing heterotrophs together with autotrophs was spiked with different concentrations of leachates (Focardi et al., 2022). Oceanospirialles were recently described to contribute substantially to PETase abundance in the mesopelagic ocean (Alam et al., 2020). PETase is a newly‐evolved hydrolase able to degrade polyethylene terephthalate (PET) plastic (Yoshida et al., 2016), and has been recently postulated as an important plastic‐degrading enzyme in the marine environment (Alam et al., 2020). In lake water communities, other genera affiliated with Gammaproteobacteria (Acinetobacter), Bacilli (*Exiguobacterium*) and Alphaproteobacteria (*Brevundimonas)* dominated the response to LDPE leachates (Sheridan et al., 2022). In other studies looking at bacteria colonizing marine plastic debris, members of the *Rhodobacteraceae* were also found to be abundant (Bos et al., 2023; Bryant et al., 2016; Oberbeckmann et al., 2021), highlighting the role that this group may play in the degradation of ocean plastic.

Besides the important contribution of generalist taxa to the leachate‐associated bacterial communities, some taxa responded specifically to certain leachates (Figures 5 and 6, Table S1). This was particularly remarkable in the irradiated aged plastic leachate treatment, where non‐generalist taxa accounted for substantial bacterial numbers, and in the non‐irradiated aged plastic leachate treatment, where they contributed up to 30% of the community. Among the non‐generalist taxa responding to leachates (Figure 5B), *P. shioyasakiensis* SE3, and Flavobacteriales 99.46% similar to *Allomuricauda aquimarina* were the main contributors. Members of the flavobacteriales have been also reported to contain PETase motifs in a study in the global ocean (Alam et al., 2020) and were recently associated with high levels of LDPE leachates use in lake waters (Sheridan et al., 2022), suggesting they may play an important role in plastics degradation. It could be that some bacteria able to degrade plastic leachates were the same degrading plastic itself, but more research should be done to asses it. Groups that dominated in the in situ communities, such as SAR11, experienced an important decline in abundance in all treatments. This was also observed in marine communities exposed to PVC leachates (Focardi et al., 2022) and during other manipulation experiments (Sebastián et al., 2021). SAR11 are slow‐growing bacteria (Kirchman, 2016) and have streamlined genomes (Giovannoni, 2017), and as such, they are usually outcompeted by fast‐growing taxa.

The differences in taxonomic composition observed between irradiated and non‐irradiated treatments (Figure 4, Table S1) suggest a change in the chemical composition of the leachates produced under irradiation, as observed before (Romera‐Castillo, Birnstiel, et al., 2022). Sunlight radiation is the main factor degrading plastic in nature (Andrady, 2011) enhancing its leaching in seawater (Romera‐Castillo, Birnstiel, et al., 2022; Romera‐Castillo, Mallenco‐Fornies, et al., 2022). Similar to the natural DOM (Kieber et al., 1997; Mopper et al., 2015), the material released by plastic is subjected to photochemical reactions giving rise to different molecules. Moreover, depending on the manufacturer, each type of plastic presents a different composition of additives that can be leached. Although the exact chemical composition of our leachates is unknown, the optical properties of the DOM were different, and aged plastic leachates presented two fluorescent peaks in the protein‐like region that were absent in the LDPE leachates (Romera‐Castillo, Birnstiel, et al., 2022). This and the total amount of DOC released could explain the differences in the microbial taxa selected in each of the plastic leachates. The differences found between irradiated and non‐irradiated treatments should be taken into account in future strategies of plastic waste treatments.

Finding the marine bacterial species able to degrade plastic leachates could help to design bioremediation measures to reduce the contaminant compounds released during plastic degradation in marine waters that can be toxic for marine life. Our work adds to the growing body of studies showing that plastic leachates produce changes in the activity and community structure of microbial communities, but goes a step further, shedding light on taxa that could play an important role in plastic leachate degradation in the marine environment. Sunlight degradation in combination with the use of the right bacterial taxa biodegrading plastic leachates could be a promising strategy for plastic chemical pollution bioremediation in the future.

## AUTHOR CONTRIBUTIONS

**Cristina Romera‐Castillo:** Conceptualization (equal); data curation (equal); formal analysis (equal); funding acquisition (lead); investigation (equal); methodology (equal); project administration (lead); supervision (equal); validation (equal); writing – original draft (equal); writing – review and editing (equal). **Stéphanie Birnstiel:** Formal analysis (equal). **Marta Sebastián:** Conceptualization (equal); data curation (equal); formal analysis (equal); investigation (equal); methodology (equal); supervision (equal); validation (equal); writing – original draft (equal); writing – review and editing (equal).

## CONFLICT OF INTEREST STATEMENT

The authors declare no conflicts of interest.

## Supporting information


**Data S1.** Supplement figures.

**Table S1.** Taxonomic affiliation (SILVA v.138) of the most‐responsive bacterial taxa (ASVs) in the leachates degradation experiment. The column cluster represents the clusters delineated in Figure 4 of main text. The closest match to an isolated type strain and its corresponding NCBI accesion number is also shown.

## Data Availability

Data are included in the article and supplementary material.
