# Supplementary material for: Diversity of marine bacteria growing on leachates from virgin and weathered plastic: Insights into potential degraders
Source: Environ Microbiol Rep. 2024 Jun 23;16(3):e13305. doi: 10.1111/1758-2229.13305 (PMC11194452; doi:10.1111/1758-2229.13305)
Supplement: Supplementary file 1 — Data S1. Supplement figures. [file EMI4-16-e13305-s001.docx]

Supporting information

**Diversity of marine bacteria growing on leachates from virgin and weathered plastic: insights into potential degraders**

Romera-Castillo, C.^1^, Birnstiel, S.^1^, Sebastián, M.^1^

^1^Instituto de Ciencias del Mar-CSIC, Barcelona, Spain

This file contains three supplementary figures. Table S1 is presented in a different excel file.


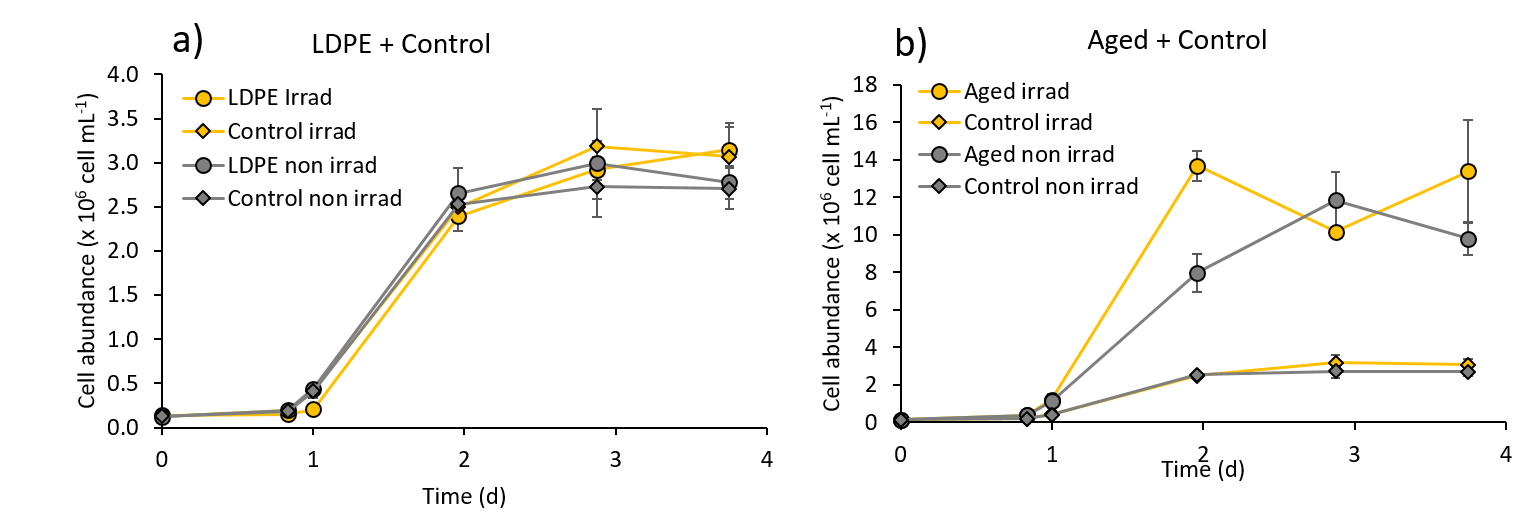


**Figure S1**. Growth of natural bacterial communities on a) LDPE leachates and b) aged plastic leachates produced during the abiotic degradation experiment (irradiated and non-irradiated). The controls with no plastics are also shown. Error bars represent the standard deviation of the mean of the triplicate cultures. Note the different scales of cell abundance in panels a) and b). From Romera-Castillo et al., 2022a.

**Figure S2.** Relative abundance of the different clusters delineated in figure 5, which group ASVs with similar trends in the different treatments, to total community sequences in each of the treatments. Colors represent the different clusters.


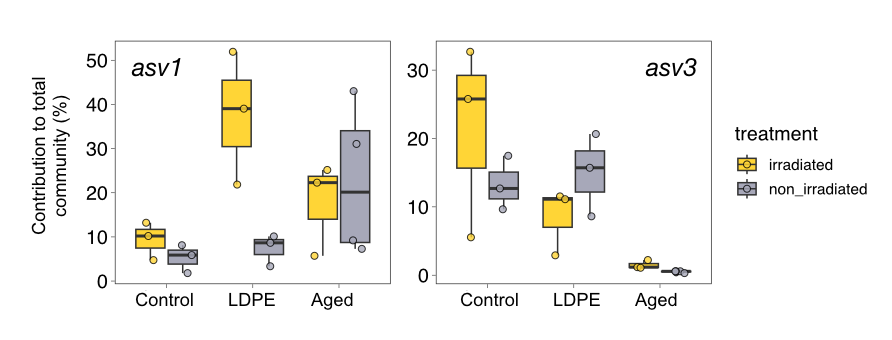


**Figure S3.** Behaviour of two generalist taxa (belonging to cluster 1, defined in Figure 5 of main document) in the different leachates. The Y-axis represents the percent contribution of the cluster to the total community in each of the samples.

Figure S4. Phylogenetic placement of ASVs related to *Pseudoalteromonas* that responded in the irradiated aged plastic leachates (cluster 4) and both irradiated and non-irradiated aged plastic leachates (cluster 3). Cluster were defined in Figure 5 of the main document. Alignment and phylogenetic reconstructions were performed using the function "build" of ETE3 3.1.2 ([Huerta-Cepas et al., 2016](https://pubmed.ncbi.nlm.nih.gov/26921390)) as implemented on the [GenomeNet (https://www.genome.jp/tools/ete/)](https://www.genome.jp/tools/ete/). ML tree was inferred using RAxML v8.2.11 ran with model PROTGAMMAJTT and default parameters ([Stamatakis, 2014](https://pubmed.ncbi.nlm.nih.gov/24451623)). Branch supports were computed out of 100 bootstrapped trees.


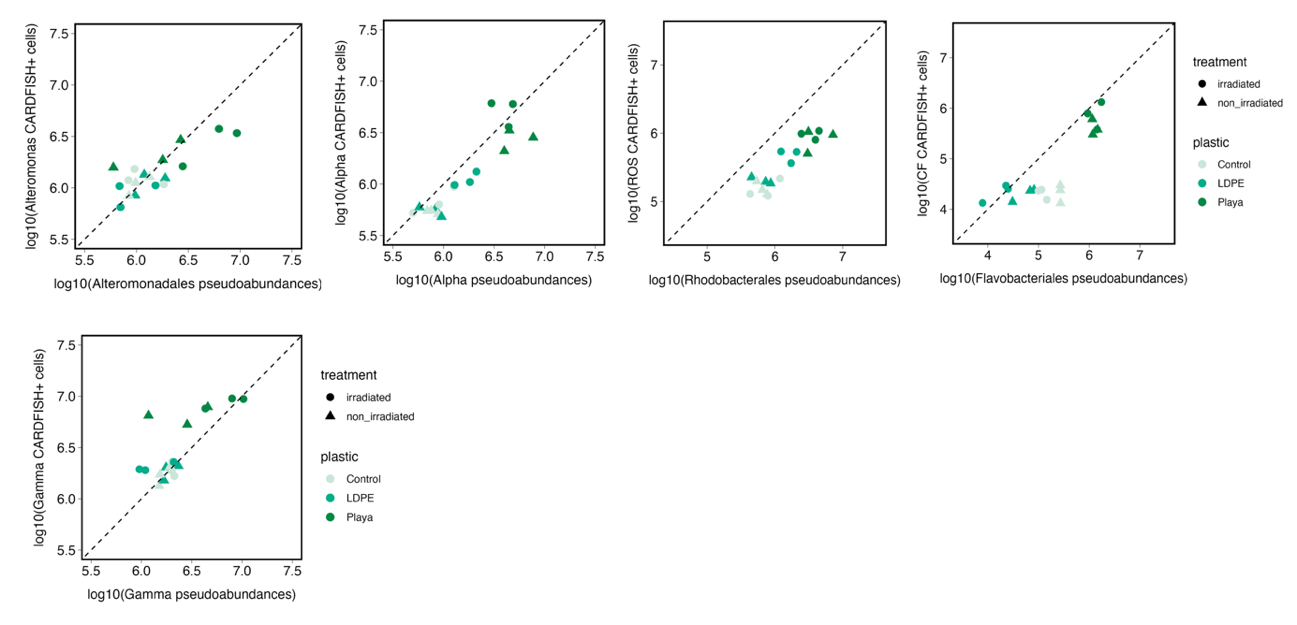


**Figure S5.** Comparison of cells abundances estimated through CARDFISH and pseudoabundances calculated by multiplying the relative 16S rRNA gene abundance of each taxonomic group with the total prokaryotic abundance obtained through flow cytometry. Samples were collected on day 4 of the experiment.
